# Coumarins, Xanthones and Related Compounds

**DOI:** 10.3390/molecules21030341

**Published:** 2016-03-10

**Authors:** Pascal Richomme

**Affiliations:** EA921 SONAS, SFR4207 QUASAV, Campus du vegetal, University of Angers, 49070 Beaucouzé, France; pascal.richomme@univ-angers.fr; Tel.: +32-249-180-437; Fax: +32-412-266-634

It has long been known that coumarins (α-pyrones) and xanthones (γ-pyrones) together form a large class of naturally occurring compounds exhibiting a wide range of biological activities. However, the interest of the scientific community for these secondary metabolites, as well as for their structural analogues, has never decreased since these “old compounds” are constantly generating promising—and sometimes unexpected—therapeutic perspectives [1]. For instance, Vadimezan [ASA404 or (5,6-dimethyl-9-oxo-9*H*-xanthen-4-yl)-acetic acid] was recently developed as a tumor vascular-disrupting agent [2] whereas many coumarin derivatives are under investigation for their anti-oxidant and anti-inflammatory properties [3]. 

In this special issue Yang *et al.* [4] investigated the blood-brain barrier permeability of 12 simple coumarins found in dried roots of *Angelicae pubescentis* used in Traditional Chinese Medicine to enrich blood, promote blood circulation and modulate the immune system. Okuyama *et al.* [5] showed that auraptene, an *O*-geranyl coumarin generally identified in *Citrus* species, directly exerts anti-inflammatory effects on mouse brain through suppression of inflammatory mediators derived from astrocytes. Using the coumarin skeleton as a starting point to design potent structural analogues Salem *et al.* [6] have synthetized new antioxidant derivatives exhibiting antitumor properties as well as protective effects against DNA damages. Coumarins may also be used as interesting scaffolds for alternative crop protections. This is the reason why Araniti *et al.* [7] explored the phytotoxic potential and biological activity of three synthetic coumarin derivatives as new natural-like herbicides whereas, in a related study, Garcia *et al.* [8] nicely described synergy and other interactions in evidence between polymethoxyflavones obtained from *Citrus* by-products.

In relation with this class of secondary metabolites different example of analytical developments are also given in this special issue. Indeed, in the aim of pharmacokinetics studies Zeng *et al.* [9] validated a LC-MS^n^ method allowing to quantify scopoletin in rat plasma whereas Medeiros-Neves *et al.* [10] focused on the quantification of coumarins in an aqueous extract of *Pterocaulon balansae.* During the latter study the main coumarin, 5,6-dimethoxy-7-(3′-methyl-2′,3′-dihydroxybutyloxy)coumarin, was described for the first time in *P. balansae* together with a new compound, namely 5,6-dimethoxy-7-(2′,3′-epoxy-3′-methylbutyloxy)coumarin. Through a dereplication analysis, seven known *Mammea* coumarins were identified by Dang *et al.* [11] in a fraction obtained from a *Mammea neurophylla* dichloromethane bark extract. Among them, examination of the NMR dataset of pedilanthocoumarin B led to a structural revision. Additionally, careful inspection of LC-DAD-MS^n^ profiles allowed the authors to predict the presence of four new compounds, which were further isolated and identified as two benzoyl substituted 4-phenylcoumarins (iso-pedilanthocoumarin B and neurophyllol C) and two 4-(1-acetoxypropyl)coumarins cyclo F (ochrocarpins H and I).

As far as xanthones are concerned two research teams focused on prenylated derivatives and their biological activities. Xia *et al.* [12] described twenty-three derivatives, including the new cowaxanthones G and H, from the leaves of *Garcinia cowa,* and studied their ability to induce cell cycle arrest, apoptosis, and autophagy in cancer cells. A chemical investigation of a methanol extract obtained from *Cudrania tricuspidata* roots led Quang *et al.* [13] to isolate nine prenylated xanthones and seven flavonoids. These prenylated xanthones showed stronger Protein Tyrosine Phosphatase 1B inhibitory effects than the flavonoids, suggesting that they may be promising targets for the future discovery of novel inhibitors, some of them being noncompetitive. On the other hand, nonprenylated xanthones were identified by Waltenberger *et al.* [14] in bitter Gentianaceae species as novel, non-toxic vascular smooth muscle cells proliferation inhibitors, which might contribute to the development of new therapeutic applications to combat restenosis. Finally, Le Pogam and Boustie [15] nicely reviewed the last developments in related lichen studies since most generally xanthones from lichen sources exhibit unique substitution patterns.

However, the interest in xanthones and coumarins is not restricted to biological activities since coumarins, as an example, may constitute a molecular model of choice to study hydrogen bonding, in the electronic excited state [16], or may also be associated with the photovoltaic performances of specific dyes [17]. To explore the physical properties of coumarins and assess their potential use as encapsulation vehicles for hydrophobic drugs, Ruiz *et al.* [18] analyzed the photophysical behavior and rotational-relaxation dynamics of a model compound in nonionic micellar environments. Finally, an optical data storage was successively performed by Gindre *et al.* [19], with various thin polymer films containing coumarin-based derivatives, as an interesting alternative to magnetic hard drives and high capacities flash memories.
